# Better decision making practices and processes.

**DOI:** 10.23889/ijpds.v7i3.1955

**Published:** 2022-08-25

**Authors:** Felicity Flack, Carolyn Adams, Judy Allen

**Affiliations:** 1 Population Health Research Network; 2 Macquarie University; 3 University of Western Australia

## Objectives

Existing decision-making practices and processes for sharing linked data for research are not keeping pace with the data tsunami and technological advances. The objectives of this project were to review existing approaches to decision making and to make recommendations for better decision-making practices and processes.

## Approach

We used a hypothetical research application to compare decision-making practices and processes for sharing linked health data for research in three jurisdictions, Western Australia, Manitoba and Scotland. to We considered the decision makers; the relevant law, policy, and guidelines; and the ethical review process to assess practice and process against metrics of good decision making - efficiency, transparency, accountability and community participation. An analysis of the similarities and differences identified common problems and challenges with existing decision-making processes. Recommendations on how to address these common problems were proposed

## Results

There were significant similarities in the decision-making processes in the three jurisdictions. These included:

- formal application processes;
- a statutory basis for decision making;
- criteria for waiving consent including low risk, impracticality, necessity, and
- protection of privacy and confidentiality; and
- at least some community participation in decision making and research.

The main areas where decision making could be improved were:

- Efficiency — the number of decision makers and duplication of the issues considered by different decision makers.
- Separation of decision making on governance criteria and ethics criteria
- criteria for waiving consent including low risk, impracticality, necessity, and
- Community involvement

## Conclusion

This project has identified several areas where decision-making about sharing linked data for research could be improved. Six internationally relevant recommendations for better decision-making were developed covering a range of issues from identifiability to community involvement.

